# Neuron-to-glia signaling drives critical period experience-dependent synapse pruning

**DOI:** 10.1038/s41598-025-11528-3

**Published:** 2025-07-16

**Authors:** Nichalas Nelson, Kendal Broadie

**Affiliations:** 1 Department of Biological Sciences, Vanderbilt University and Medical Center, Nashville, TN 37235 USA; 2 Department of Cell and Developmental Biology, Vanderbilt University and Medical Center, Nashville, TN 37235 USA; 3 Department of Pharmacology, Vanderbilt University and Medical Center, Nashville, TN 37235 USA; 4 Kennedy Center for Research on Human Development, Vanderbilt University and Medical Center, Nashville, TN 37235 USA; 5 Vanderbilt Brain Institute, Vanderbilt University and Medical Center, Nashville, TN 37235 USA

**Keywords:** Phosphatidylserine, Draper/MEGF10, Insulin-like peptide, Insulin receptor, Cell biology, Neuroscience

## Abstract

Critical periods enable early-life synaptic connectivity optimization whereby initial sensory experience remodels circuits to a variable environment. In the *Drosophila* juvenile brain, synapse remodeling occurs within the precisely-mapped olfactory circuit, which has an extensively characterized, manageably short (< 1 week) critical period. In this brain circuit, single receptor olfactory sensory neuron (OSN) classes synapse onto single projection neurons extending to the central mushroom body learning/memory center. Critical period odorant experience drives OSN synapse remodeling, which can only be reversed during this brief interval. Our objective is to dissect intercellular signaling pathways from neurons to glial phagocytes sculpting synapse elimination in response to critical period experience. We find critical period experience causes externalized phosphatidylserine (PS) exposure in activated OSN synaptic glomeruli in an experiential dose-dependent mechanism. We discover that genetic knockdown of phosphatidylserine synthase inhibits critical period experience-dependent pruning of these synaptic glomeruli. We show a genetic interaction in *trans*-heterozygous mutants of phosphatidylserine synthase and Draper (mammalian MEGF10), the well-conserved glial engulfment receptor that binds phosphatidylserine, with double *trans*-heterozygotes blocking critical period experience-dependent pruning. This interaction mechanistically links phosphatidylserine signaling to glial phagocytosis synapse elimination. We identify the OSN scramblase that transports phosphatidylserine from the synaptic membrane inner to outer leaflet, and demonstrate phosphatidylserine externalization is rate-limiting for experience-dependent synaptic glomeruli pruning. We discover glial insulin receptors direct experience-dependent glial infiltration phagocytosis. We find activated glial insulin receptor signaling elevates critical period synapse pruning. Together this work identifies coupled intercellular signaling pathways from target neurons to glial phagocytes orchestrating experience-dependent synapse elimination.

## Introduction

 Critical periods are important early-life epochs during which neural circuit synapse connectivity is strongly remodeled by sensory input to adapt animals to an unpredictable environment^1^. These intervals open with the onset of sensory experience and close after stabilization forces prevent further large-scale synaptic wiring changes^2–4^. Critical periods enable structural and functional circuit changes in both mammals and *Drosophila*, with experience-dependent remodeling impairments in human neurodevelopmental disorders. As in mammals, *Drosophila* exhibit temporally-restricted critical periods^5–7^. In the olfactory circuit, synaptic innervation of the antennal lobe in the juvenile *Drosophila* brain is strongly remodeled in response to odorant input during a critical period following eclosion^8–10^. The Or42a receptor olfactory sensory neuron (OSN) class innervating ventral medial 7 (VM7) synaptic glomeruli exhibits striking synaptic pruning following exposure to the odorant ethyl butyrate (EB)^11,12^. This experience-dependent remodeling occurs only in the critical period, and is completely reversible in this window^11,12^. We previously discovered that this experience-driven critical period synapse elimination is mediated by glial phagocytosis in a temporally-restricted, transiently-reversible, and dose-dependent mechanism^13–16^. We found early-life EB experience drives glia to infiltrate the VM7 synaptic glomeruli and utilize the highly conserved Draper-Basket-Cheerio (mammalian MEGF10-JNK-FLNA) signaling pathway to phagocytose targeted Or42a OSN synapses^13^. This work suggests than experience-dependent intercellular signaling between these specific synapses and infiltrating glial phagocytes directs critical period synaptic pruning.

Synapse elimination by brain glia requires cell-surface and secreted signals^1,17,18^. Experience-targeted synapses present local membrane ligands that bind glial receptors to initiate engulfment and phagocytosis^17^. The membrane lipid phosphatidylserine (PS) produced by a dedicated PS synthase (PSS) is moved from the inner to outer leaflet by an activated transporter to serve as a phagocytic signal^19^. Transgenic reporters such as the membrane-associated pH sensor (MApHS)^20^ can visualize glial phagocytosis and PS-binding lactadherin (LactC1C2)^20,21^ can monitor the PS externalization. Such signaling mediates both mammalian retinogeniculate synapse refinement and *Drosophila* neuronal pruning^20,22,23^. The highly conserved glial Draper/MEGF10 receptor binds directly to the PS ligand^24^ to mediate the glial phagocytosis of developmentally-transient neurons and debris clearance in injury models^25,26^. We previously discovered that experience-driven critical period Or42a OSN synapse elimination is mediated by the glial Draper/MEGF10 receptor^13^. Membrane PS externalization has been demonstrated through the action of the *Drosophila* Subdued scramblase in vitro^27^ and can be forced via the over-expression (OE) of the mammalian homologue transmembrane protein 16 F (TMEM16F)^20^. Secreted signals work in parallel to direct glial synapse pruning^1^. The highly conserved glial insulin receptor (InR) binds neuronal insulin-like peptides (ILPs) for phagocytosis of *Drosophila* developmentally-transient brain neurons, and also brain neuronal debris clearance in injury models^25,28,29^. Our objective was to test these signaling mechanisms in experience-dependent synapse elimination during the early-life olfactory critical period.

Here, we use the Or42a OSN experience-dependent synapse pruning model^14^ to genetically dissect neuron-to-glia signaling mechanisms enabling critical period olfactory circuit remodeling. We find temporally-restricted olfactory experience during the early-life critical period drives exposure of externalized phosphatidylserine in synaptic glomeruli in a dose-dependent mechanism, and that phosphatidylserine synthase function is required for experience-driven synaptic glomeruli pruning. EB exposure-dependent innervation pruning occurs only within the short critical period and is totally reversible if the olfactory experience is eliminated during this interval^11,12^. We discover a tight genetic interaction between phosphatidylserine synthase and glial Draper engulfment receptors, with double *trans*-heterozygous mutants completely blocking all critical period synapse elimination in response to experience. We find the Subdued scramblase acting in the Or42a olfactory neurons is required for experience-driven synaptic glomeruli pruning, and that exogenous TMEM16F scramblase over-expression accelerates synapse elimination in response to lowered levels of critical period experience. These results indicate that phosphatidylserine transport from the inner to outer membrane leaflet is rate-limiting for synapse pruning. Finally, we discover glial insulin receptor function is required for experience-dependent infiltration phagocytosis, and that constitutively active InR signaling elevates the extent of glial synapse elimination in response to critical period experience. In combination, these findings reveal multiple experience-dependent mechanisms that drive local neuron-to-glia signaling to enable targeted critical period synapse elimination in the juvenile brain.

## Results

### Experience drives temporally-restricted pruning with dose-dependent PS externalization

**Fig. 1:**
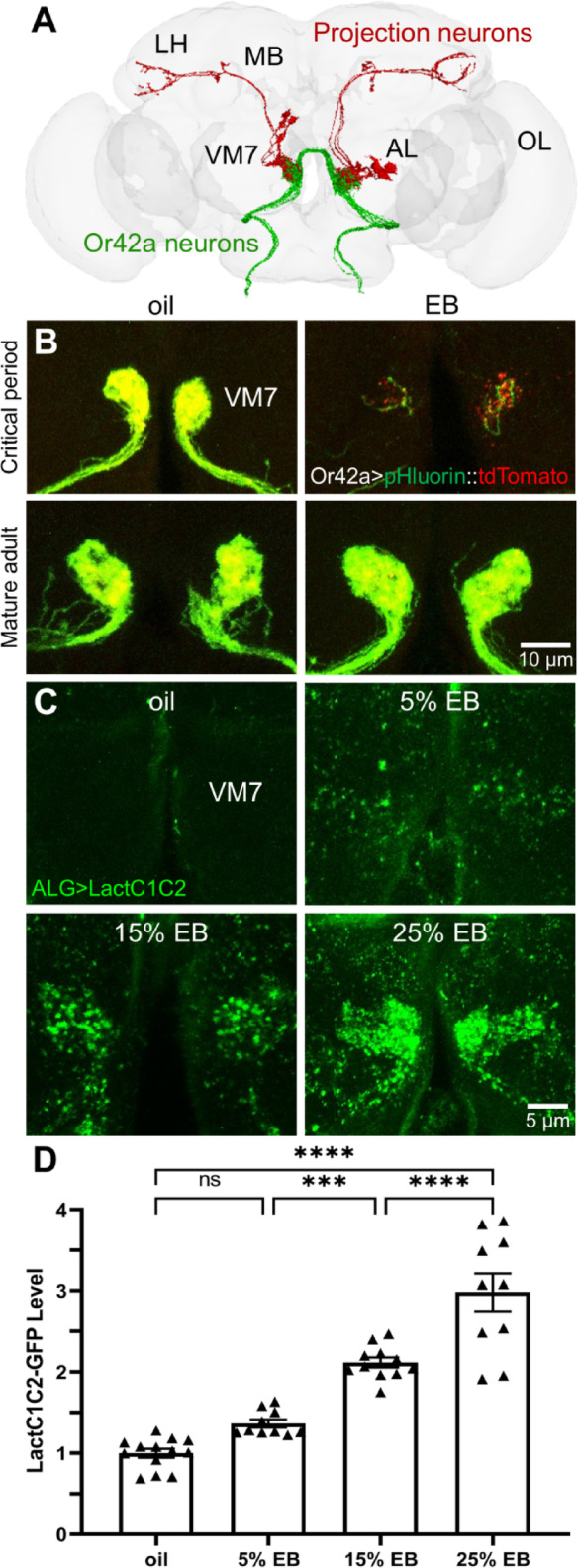
Critical period odorant experience triggers synaptic PS externalization. (**A**) Brain connectome schematic of the EB-responsive Or42a olfactory sensory neurons synapsing onto projection neurons within VM7 glomeruli (https://flywire.ai). Abbreviations: the antennal lobe (AL), mushroom body (MB), lateral horn (LH), and the optic lobe (OL). (**B**) Representative images of Or42a neurons innervating VM7 synaptic glomeruli with *Or42a*-Gal4 driven UAS-MApHS-pHluorin::tdTomato after 24-hour exposure to oil vehicle control (oil, left) or ethyl butyrate odorant (EB, right). Experience was applied either during the critical period (0–1 days post-eclosion (dpe); top) or in mature adults (7–8 dpe; bottom). Synaptic glomeruli pruning (red) is temporally-restricted within the critical period (top). Scale bar: 10 μm. (**C**) Representative images of VM7 glomeruli labeled for astrocyte-like glia (ALG)-Gal4 driven UAS-LactC1C2::GFP (*ALG > LactC1C2*) following 24-hour critical period exposure to the oil vehicle (top left), 5% EB (top right), 15% EB (bottom left), and 25% EB (bottom right). Synaptic glomeruli PS externalization (green) is dose-dependent. Scale bar: 5 μm. (**D**) Quantification of LactC1C2-GFP fluorescence intensity within the VM7 glomeruli normalized to the oil vehicle control, compared to 5–25% EB. Scatterplots show all the data points and the mean ± SEM. Significance indicated as not significant (*p* > 0.05, ns), or significant at *p* ≤ 0.001 (***) and *p* ≤ 0.0001 (****).

The *Drosophila* olfactory circuit is an excellent model to test experience-dependent synaptic pruning in a well-defined critical period^5,8,12^. Or42a receptor OSN neurons project axons that innervate VM7 glomeruli in the central brain antennal lobe (Fig. 1A). These neurons synapse on projection neurons, which extend to the mushroom body (MB) and lateral horn (LH; Fig. 1A)^30,31^. Using *Or42a*-Gal4 to drive a membrane-associated pH sensor (MApHS)^20,32^ allows the direct imaging of synapse phagocytosis (Fig. 1B). The pH-sensitive membrane pHluorin alongside the pH-insensitive tdTomato reveals the pHluorin signal diminishing relative to tdTomato as pH decreases during phagocytosis. During the early-life critical period, 24-hour exposure at 0–1 days post-eclosion (0–1 dpe) to a mineral oil vehicle reveals the normal VM7 synaptic glomeruli innervation (Fig. 1B, top left), whereas the same exposure to ethyl butyrate (EB) odorant (25%, v/v in the oil) results in striking phagocytic pruning of synaptic glomeruli innervation (Fig. 1B, top right). To test if this synaptic elimination is restricted to the critical period, mature adults (7 dpe), well outside of the critical period^11,12,15^were similarly exposed to the vehicle control and EB experience (Fig. 1B, bottom row). Control 24-hour oil vehicle exposure (7–8 dpe) shows the normal Or42a OSN innervation (Fig. 1B, bottom left), which is not altered by EB experience (Fig. 1B, bottom right). Thus, experience-dependent phagocytic pruning of synaptic glomeruli innervation is temporally-restricted to the critical period.

Glial phagocytes can recognize externalized phosphatidylserine (PS)^20,22^. A secreted LactC1C2::GFP transgenic reporter that selectively binds external PS^20,33^ was expressed in astrocyte-like glia to test EB experience-dependent signaling (Fig. 1C). In 24-hour (0–1 dpe) oil vehicle controls, LactC1C2::GFP shows little detectable PS in the VM7 synaptic glomeruli (Fig. 1C, top left). In contrast, the same period of EB experience causes a dose-dependent increase in externalized PS, with 5% EB resulting in a faint signal specifically concentrated at VM7 glomeruli (Fig. 1C, top right), 15% EB a strikingly punctate PS distribution (Fig. 1C, bottom left), and 25% EB producing strong PS widely in VM7 (Fig. 1C, bottom right). Quantification of VM7 fluorescence intensity normalized to oil vehicle control (1.0 ± 0.053 (mean ± SEM), *n* = 13) shows slight, but not significant, increased signal with 5% EB (1.366 ± 0.049, *n* = 10; *q*_(40)_ = 3.224, *p* = 0.120; Fig. 1D, left), a stronger increase at 15% EB (2.117 ± 0.061, *n* = 11) significantly elevated compared to 5% EB (*q*_(40)_ = 6.358, *p* = 3.3 × 10^−4^; Fig. 1D, center), and the strongest increase at 25% EB (2.982 ± 0.023, *n* = 10) significantly elevated compared to 15% EB (*q*_(40)_ = 7.327, *p* = 3.8 × 10^−5^; Fig. 1D, right). A one-way ANOVA with Tukey’s multiple comparison post-hoc test comparing the vehicle vs. differing EB concentrations shows significant effects for all experiences (*F*_(3,40)_ = 57.69, *p* = 1.4 × 10^−14^). Taken together, these findings demonstrate that the Or42a OSN synaptic glomeruli specifically externalize PS in an EB dose-dependent experience mechanism during the early-life critical period.

### Phosphatidylserine synthesis is essential for experience-dependent synapse pruning

**Fig. 2:**
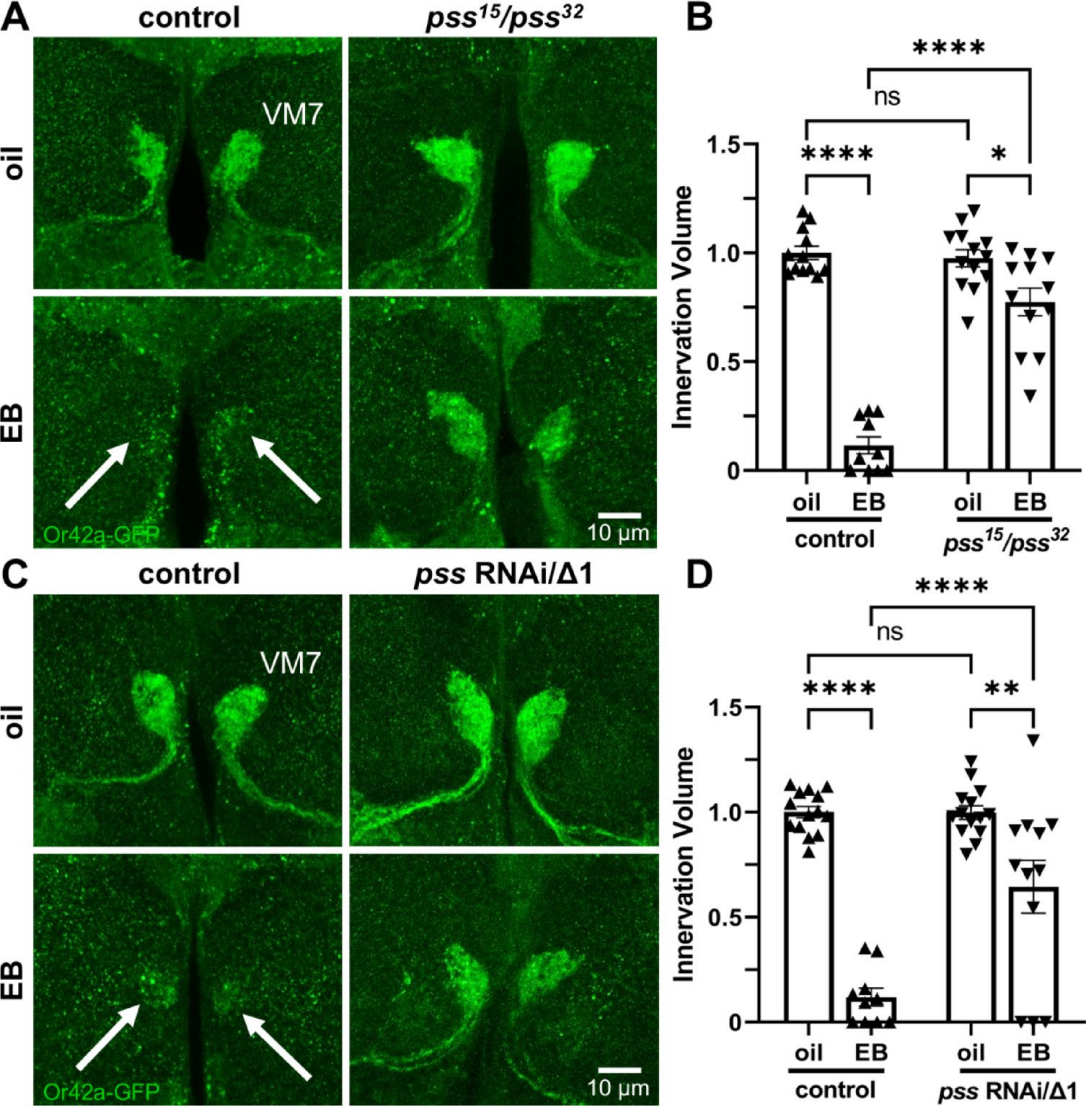
Phosphatidylserine synthase is critical for experience-dependent pruning. (**A**) Representative images of Or42a neurons innervating VM7 synaptic glomeruli with the *Or42a* promoter driven UAS-mCD8::GFP membrane marker (*Or42a*-GFP, green) after 24-hour critical period (0–1 dpe) exposure to oil vehicle (top) or EB experience (bottom). The *Or42a*-GFP/+ genetic background control (left) shows robust experience-dependent synaptic glomeruli pruning (arrows), which fails in *trans*-heterozygous *pss*^*15*^/*pss*^*32*^ mutants (right). Scale bar: 10 μm. (**B**) Quantification of the Or42a OSN innervation volume in VM7 glomeruli in all conditions, normalized to the oil vehicle control. (**C**) Representative images under the same conditions as above with the *Or42a*-GFP/+ genetic background control (left) compared to targeted *Or42a*-Gal4 driven UAS-*pss* RNAi in *pss*^*∆1*^/+ heterozygotes (*pss* RNAi/*pss*^*∆1*^, right). Scale bar: 10 μm. (**D**) Quantification of the Or42a OSN innervation volume within VM7 glomeruli in all four conditions, normalized to the oil vehicle control. Scatterplots show all the data points and the mean ± SEM. Significance is indicated as not significant (*p* > 0.05; ns), or significant at *p* ≤ 0.05 (*), *p* ≤ 0.01 (**), and *p* ≤ 0.0001 (****).

Given Or42a synapses present PS in response to critical period EB experience, the next question is whether this phospholipid is required for synaptic glomeruli pruning. To disrupt PS production, the sole *Drosophila* enzyme responsible for PS synthesis is the conserved phosphatidylserine synthase (PSS)^34,35^. Null *pss* mutants are embryonic lethal, so two *pss* hypomorphic mutants were used in combination (*pss*^*15*^*/pss*^*32*^) to allow adult survival^34^. This viable *pss* mutant condition was compared with the *w*^*1118*^ genetic background control. Compared to mutants, *pss* RNAi is a weak PS knockdown condition^34^, so *pss* RNAi was combined with a heterozygous *pss*^Δ1^ null mutant. The *pss* RNAi was Or42a neuron-targeted with specific *Or42*-Gal4 in a *pss*^Δ1^/+ background (*pss* RNAi/∆1). To assess critical period pruning in the EB-responsive VM7 synaptic glomeruli, newly eclosed juvenile animals were exposed for 24-hours (0–1 dpe) to either a mineral oil vehicle or EB odorant dissolved in oil. The mCD8::GFP plasma membrane marker expressed specifically in the Or42a neurons (*Or42a-*GFP) was used to visualize VM7 synaptic glomeruli innervation^14^. The 3-dimension innervation volume was measured to compare the vehicle control and EB experience conditions, within and between genotypes. Representative images for the two mutant and control genotypes, and both critical period experience treatments (8 conditions), are presented with the quantification for all individual data points and mean ± SEM in Fig. 2.

In the genetic background control, the oil vehicle treatments show the normal Or42a OSN innervation of the VM7 synaptic glomeruli (Fig. 2A, top left). VM7 glomeruli are adjacent to the antennal lobe border at the ventral midline, with the Or42a OSN innervation filling each paired glomerulus. In sharp contrast, critical period EB experience causes strong pruning of the synaptic glomeruli (Fig. 2A, bottom left, arrows). The EB experience drives a striking reduction in Or42a OSN innervation, with little mCD8::GFP signal persisting in the VM7 glomeruli. The *pss* mutant (*pss*^*15*^*/pss*^*32*^) largely prevents all EB experience-dependent synaptic pruning, with the vehicle control and EB treatment conditions showing similar VM7 innervation (Fig. 2A, right column). Quantification of the Or42a OSN innervation volume normalized to the oil vehicle genetic control (1.0 ± 0.031, *n* = 12) shows that critical period EB experience causes significant innervation volume reduction (0.116 ± 0.039, *n* = 10; *q*_(43)_ = 18.89, *p* = 1.1 × 10^−12^; Fig. 2B, left). In contrast, the *pss* mutant exhibits similar innervation volumes to controls with the oil vehicle (0.975 ± 0.039, *n* = 13), and only slight Or42a OSN innervation pruning with critical period EB experience (0.774 ± 0.063, *n* = 12; *q*_(43)_ = 4.589, *p* = 0.012; Fig. 2B, right). A two-way ANOVA (2 × 2) with Tukey’s multiple comparison post-hoc test comparing the innervation volumes reveals significant effects for both genotype (*F*_(1,43)_ = 48.84, *p* = 1.3 × 10^−8^) and odorant exposure (*F*_(1,43)_ = 143.3, *p* = 3.0 × 10^−14^), with significant interaction between genotype and experience (*F*_(1,43)_ = 56.83, *p* = 2.1 × 10^−9^; Fig. 2B).

We next tested PSS knockdown in the Or42a neurons as best possible, with Or42a OSN-targeted *pss* RNAi in the *pss*^Δ1^/+ null background. The driver control (*Or42a*-Gal4/+) shows normal innervation of the VM7 synaptic glomeruli in the oil vehicle control condition (Fig. 2C, top left). In contrast, 24-hour critical period EB experience (0–1 dpe) causes striking synaptic pruning (Fig. 2C, bottom left, arrows). The Or42a neuron-targeted *pss* RNAi clearly inhibits this EB experience-dependent pruning, albeit with a small degree of synapse elimination persisting (Fig. 2C, right column). Tukey’s multiple comparison tests normalized to oil vehicle driver control innervation volume (1.0 ± 0.027, *n* = 14) reveals significant pruning with critical period EB exposure (0.119 ± 0.043, *n* = 10; *q*_(46)_ = 12.78, *p* = 5.5 × 10^−11^; Fig. 2D, left). The targeted *pss* RNAi; *pss*^Δ1^/+ mutant is similar to the driver controls with oil vehicle exposure (0.998 ± 0.032, *n* = 14), but shows significant impairment in the Or42a innervation synaptic glomeruli pruning following 24-hour critical period EB experience (0.645 ± 0.125, *n* = 12; *q*_(46)_ = 5.393, *p* = 0.002; Fig. 2D, right). A two-way ANOVA (2 × 2) with Tukey’s multiple comparison post-hoc test comparing the innervation volumes reveals significant effects for both genotype (*F*_(1,46)_ = 15.18, *p* = 3.1 × 10^−4^) and critical period odor (*F*_(1,46)_ = 84.20, *p* = 5.8 × 10^−12^), with a significant interaction (*F*_(1,46)_ = 15.39, *p* = 2.9 × 10^−4^; Fig. 2D). Together, these findings suggest Or42a neuron phosphatidylserine production plays a central role in EB experience-dependent synaptic pruning in the critical period.

### PS synthase and Draper receptor functions interact in critical period synaptic pruning

Given that neuronal PS production is important for proper experience-dependent synaptic glomeruli pruning (Figs. 1 and 2), we next tested for an interaction with the glial PS receptor mediating synapse engulfment phagocytosis. We previously discovered that the Draper (mammalian MEGF10) glial receptor is absolutely essential for EB experience-dependent critical period pruning of the Or42a innervation in VM7 synaptic glomeruli^13^. Draper binds PS directly^24^and the glial Draper receptor mediates phagocytic pruning in a number of contexts^25,26,36,37^often partnered with a PS adaptor^33^. To test PS ligand and Draper receptor interaction in experience-dependent synapse elimination during the critical period, we combined together two null mutants in *trans*-heterozygous combination (*pss*^Δ1^/+; *draper*^Δ5^/+). This genetic nonallelic noncomplementation test has been used repeatedly to define *Drosophila* synaptic interactions in vivo^25,38–40^. The two individual heterozygous mutants (*pss*^Δ1^/+ and *draper*^Δ5^/+) were each compared to the *trans*-heterozygote (shown as *pss*/*draper*) as well as the *w*^*1118*^ genetic background control. If disrupted experience-dependent critical period pruning were to be only observed in the *trans*-heterozygote, with no effect in the individual heterozygotes, this would indicate a direct genetic interaction, placing the gene products in the same process^25,38–40^. Or42a OSN innervation of the VM7 glomeruli was tested following early-life critical period oil vehicle or EB exposure for all four genotypes (8 conditions).

**Fig. 3:**
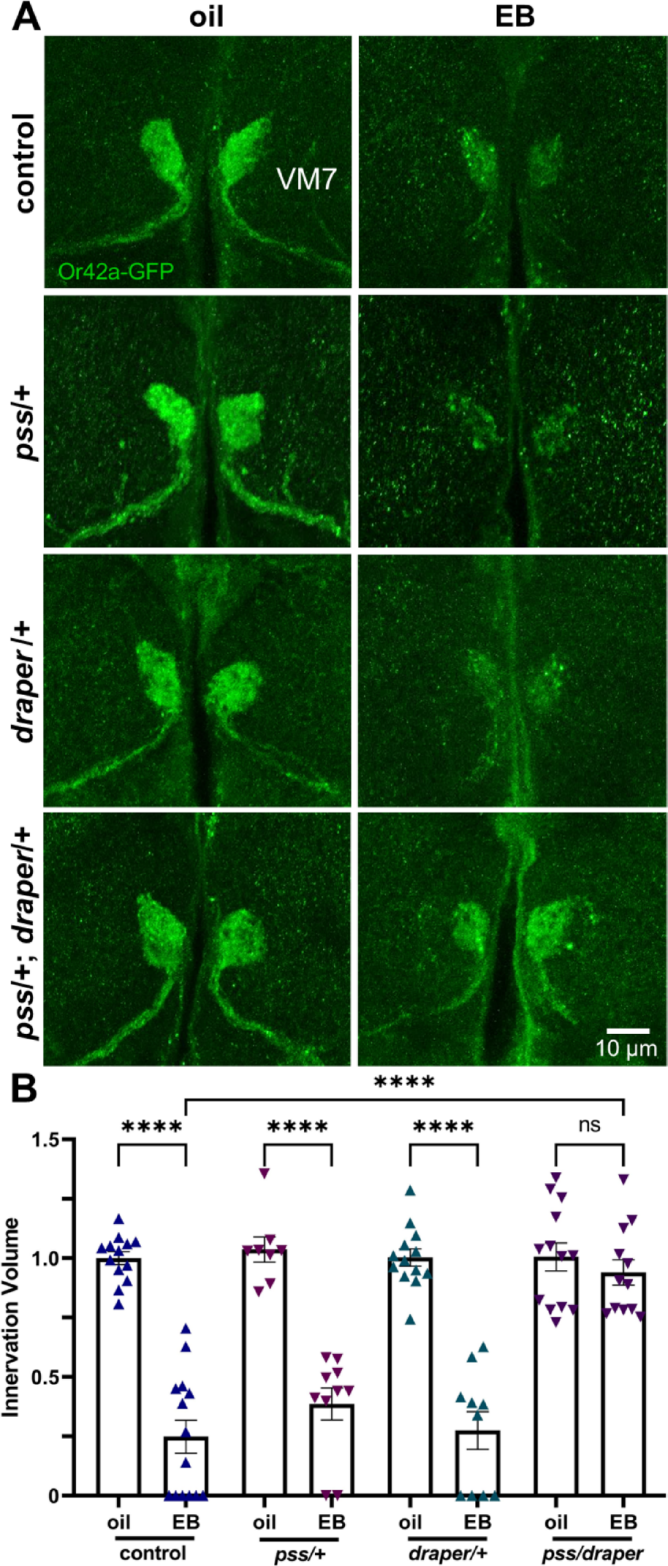
Draper and PS synthase interact for critical period synaptic pruning. (**A**) Representative images of the Or42a neuron innervation of the VM7 synaptic glomeruli (*Or42a*-GFP, green) with 24-hour critical period exposure to oil vehicle (left) or EB (right). Robust innervation pruning occurs in the genetic background control (*Or42a*-GFP/+, top). Similar VM7 synaptic glomeruli pruning occurs in *pss*^*∆1*^/+ and *draper*^*∆5*^/+ heterozygote controls comparing the oil vehicle (left) with EB experience (right, middle two rows). The double *trans*-heterozygous combination (*pss*^*∆1*^/+; *draper*^*∆5*^/+) shows normal innervation in the oil vehicle (bottom, left) and full block of experience-dependent synaptic glomeruli pruning with the EB odorant (bottom, right). Scale bar: 10 μm. (**B**) Quantification of the Or42a OSN innervation volume in VM7 glomeruli normalized to the genetic background oil vehicle control for all four genotypes and both conditions (8 comparisons). For each matched pair, 24-hour critical period exposure to oil vehicle (left) and EB odorant (right) is shown. Scatterplots show all the data points and the mean ± SEM. Significance is indicated at *p* ≤ 0.0001 (****), or not significant (*p* > 0.05; ns).

The oil vehicle controls exhibit the normal Or42a OSN innervation of VM7, and critical period EB experience causes the normal glial synaptic glomeruli pruning (Fig. 3A, top row). Both phosphatidylserine synthase (*pss*^*Δ1*^/+) and Draper receptor (*draper*^*Δ5*^/+) null heterozygotes fail to have any discernable effect on experience-dependent synaptic glomeruli pruning (Fig. 3A, middle rows). In sharp contrast, the double *trans*-heterozygote (*pss*^*Δ1*^*/draper*^*Δ5*^) completely prevents critical period synapse elimination (Fig. 3A, bottom row). Quantification shows Or42a OSN innervation volume normalized to the oil vehicle control (1.0 ± 0.027, *n* = 13) is pruned by EB experience (0.248 ± 0.069, *n* = 14; *q*_(85)_ = 9.973, *p* = 1.6 × 10^−14^; Fig. 3B, left). The *pss* heterozygote oil (1.036 ± 0.053, *n* = 8) and EB (0.386 ± 0.067, *n* = 10; *q*_(85)_ = 7.007, *p* = 1.5 × 10^−8^; Fig. 3B, center) conditions show the same degree of innervation pruning. Likewise, the *draper* heterozygote oil condition is not significantly different (1.003 ± 0.036, *n* = 13), and is similarly pruned by EB experience (0.274 ± 0.080, *n* = 10; *q*_(85)_ = 8.849, *p* = 3.1 × 10^−12^; Fig. 3B, center). In contrast, the double heterozygotes (*pss*^*Δ1*^*/draper*^*Δ5*^) show no experience-dependent pruning, with the oil vehicle innervation (1.005 ± 0.059, *n* = 13) not significantly different following EB experience (0.940 ± 0.054, *n* = 12; *q*_(85)_ = 0.840, *p* = 0.999; Fig. 3B, right) based on ANOVA analyses with a Tukey’s multiple comparison post-hoc test. These findings demonstrate that interaction between neuronal PS synthesis and the glial Draper/MEGF10 phagocytic receptor is necessary for experience-dependent synapse pruning during the critical period.

### Neuronal scramblase mediates and limits experience-dependent synapse pruning

Phosphatidylserine normally resides in the inner leaflet of the plasma membrane, but is moved to the outer leaflet when serving as a membrane surface phagocytic ligand^19,41^. Neuronal scramblase activity has been shown to mediate this PS externalization mechanism^20,42^. Given that experience-dependent PS production interacts with glial Draper phagocytic receptors to drive synapse pruning (Figs. 1, 2 and 3), we hypothesized that a neuronal scramblase is transporting PS from the inside to the outside of Or42a OSN synaptic membranes in response to critical period EB experience. The *subdued* gene product functions as an endogenous *Drosophila* PS scramblase^27^. To test mechanism, the Or42a neuron-targeted *Or42a*-Gal4 driver was used to express UAS-*subdued* RNAi (Or42a > *subdued* RNAi). Using the strong 25% EB experience condition, VM7 synaptic glomeruli pruning was assayed during the critical period. To complement this experiment, the mammalian *subdued* homolog TMEM16F PS scramblase was over-expressed in the Or42a neurons^43,44^. TMEM16F has previously been demonstrated to transport PS for neuronal membrane externalization in *Drosophila*^20,45^. In this assay, the weaker 15% EB experience condition was used to test whether VM7 synaptic glomeruli pruning is exacerbated following critical period odorant exposure. If high experience-dependent synapse pruning is impaired with scramblase loss, and there is more pruning with lowered experience upon neural scramblase over-expression, this would indicate PS scramblase externalization is rate-limiting for critical period synapse elimination.

**Fig. 4:**
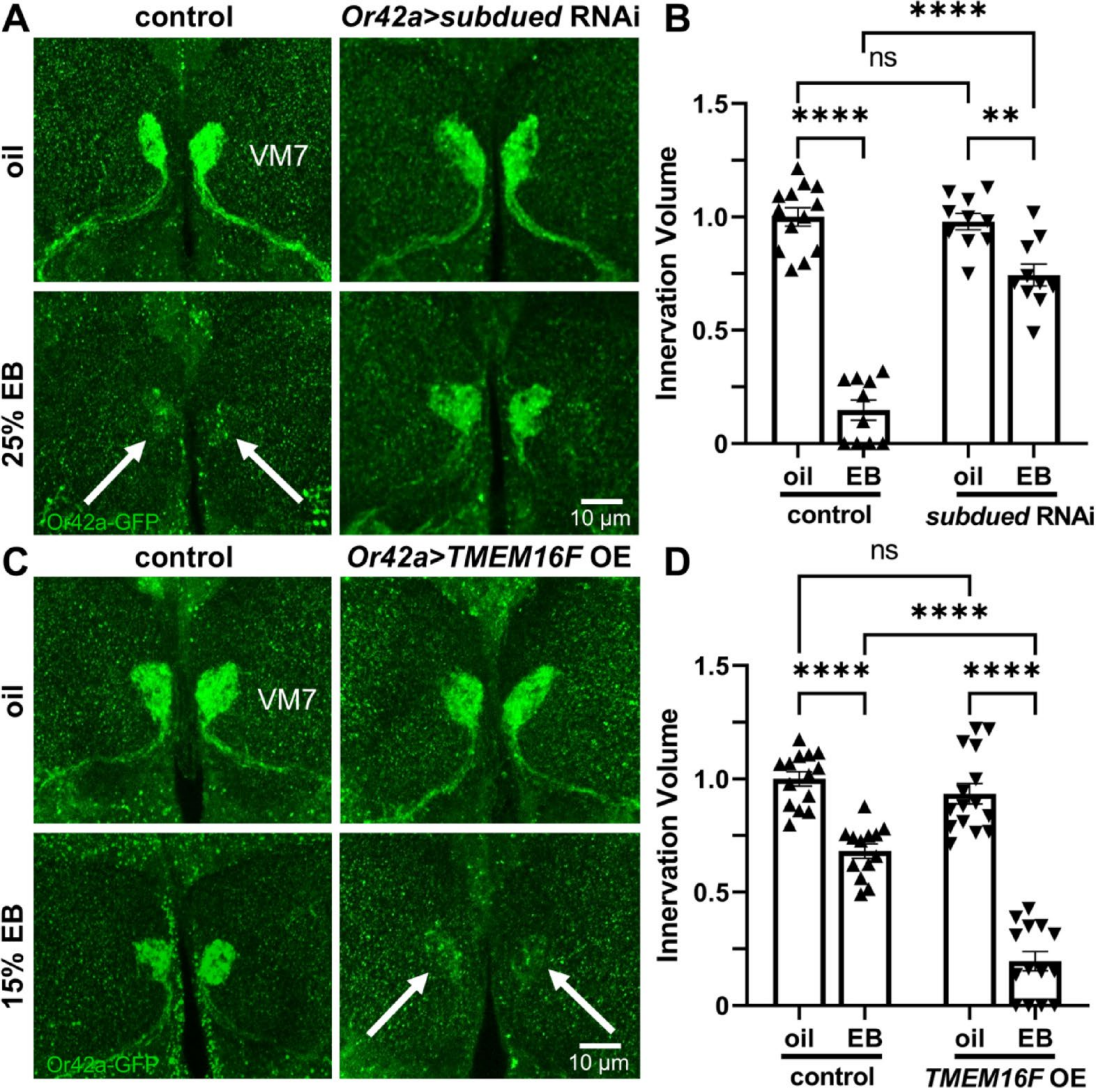
Neuronal scramblase is rate-limiting for experience-dependent pruning. (**A**) Representative images of Or42a neurons innervating the VM7 glomeruli (*Or42a*-GFP, green) following 24-hour critical period exposure to oil vehicle (top) or 25% EB odorant (bottom). Normal experience-dependent pruning occurs in driver controls (*Or42a*-Gal4/+, left, arrows), which is impaired by *Or42a*-Gal4 targeted UAS-*subdued* scramblase RNAi (*Or42a* > *subdued* RNAi, right). Scale bar: 10 μm. (**B**) Quantification of the Or42a OSN innervation volume in VM7 glomeruli in all conditions, normalized to the oil vehicle control. (**C**) Representative images under the same conditions as above with the driver control (*Or42a*-Gal4/+, left) compared to *Or42a*-Gal4 driven UAS-*TMEM16F* scramblase over-expression (*Or42a* > *TMEM16F* OE, right) in oil vehicle (top) or 15% EB (bottom). Elevated scramblase exacerbates critical period experience-dependent pruning in the reduced EB condition (bottom, right, arrows). Scale bar: 10 μm. (**D**) Quantification of the Or42a OSN innervation volume in VM7 glomeruli in all conditions, normalized to the oil vehicle control. Scatterplots show all the data points and the mean ± SEM. Significance is indicated as not significant (*p* > 0.05; ns), or significant at *p* ≤ 0.01 (**) and *p* ≤ 0.0001 (****).

To test whether endogenous neuronal scramblase activity mediates critical period experience-dependent synaptic glomeruli pruning, the Or42a neuron-targeted *subdued* RNAi was first assayed. In the transgenic driver control (*Or42a*-Gal4/+), the 24-hour oil vehicle condition (0–1 dpe) shows the normal Or42a OSN innervation of the VM7 synaptic glomeruli, and the 25% EB critical period experience causes the normal strong pruning (Fig. 4A, left column, arrows). In contrast, the Or42a neuron-targeted *subdued* RNAi very clearly inhibits this EB experience-dependent synaptic glomeruli pruning (Fig. 4A, right column). Tukey’s multiple comparison tests normalized to oil vehicle control innervation volume (1.0 ± 0.040, *n* = 13) reveals the expected highly significant innervation volume pruning with the stronger 25% EB critical period experience (0.150 ± 0.044, *n* = 10; *q*_(39)_ = 20.56, *p* = 1.3 × 10^−13^; Fig. 4B, left). In comparison, Or42a neuron-targeted *subdued* RNAi is similar to driver controls for the oil vehicle innervation volume (0.979 ± 0.036, *n* = 10), but shows very significantly less pruning of Or42a OSN innervation compared with control EB-treated animals (0.744 ± 0.048, *n* = 10; *q*_(39)_ = 13.52, *p* = 5.4 × 10^−11^; Fig. 4B, right). A two-way ANOVA (2 × 2) comparing innervation volumes shows significant effects for both genotype (*F*_(1,39)_ = 45.16, *p* = 5.2 × 10^−8^) and critical period odor exposure (*F*_(1,39)_ = 161.7, *p* = 2.0 × 10^−14^), with a significant interaction term (*F*_(1,39)_ = 51.92, *p* = 1.1 × 10^−8^; Fig. 4B). Thus, neural Subdued PS scramblase mediates experience-dependent synapse pruning.

To test if *subdued* homolog TMEM16F PS scramblase over-expression (OE) can exacerbate the synaptic glomeruli pruning, Or42a neuron-targeted UAS-*TMEM16F* OE was next assayed (*Or42a* > *TMEM16F* OE). A lower EB concentration (15%) was used to allow visualization of the potentially elevated experience-dependent synapse elimination. In the *Or42a*-Gal4/+ driver control, the oil condition shows normal Or42a OSN innervation of the VM7 glomeruli, whereas the lower 15% critical period EB experience results in the dose-dependent reduction in experience-driven synaptic glomeruli pruning (Fig. 4C, left column). In contrast, TMEM16F over-expression in Or42a neurons substantially elevates 15% EB experience-dependent pruning (Fig. 4C, right column, arrows). Quantification of innervation volume normalized to the oil vehicle control (1.0 ± 0.031, *n* = 14) shows that 15% critical period EB experience in the driver control causes a significant ~ 30% volume reduction (0.681 ± 0.032, *n* = 13; *q*_(52)_ = 8.086, *p* = 3.1 × 10^−6^; Fig. 4D, left). In comparison, the *TMEM16F* OE oil condition is normal (0.934 ± 0.045, *n* = 15), but there is a significantly larger > 80% innervation volume loss with experience (0.196 ± 0.042, *n* = 14; *q*_(52)_ = 19.43, *p* < 1.0 × 10^−14^; Fig. 4D, right). A two-way ANOVA with Tukey’s multiple comparison post-test reveals significant effects for both genotype (*F*_(1,52)_ = 50.73, *p* = 3.2 × 10^−9^) and experience (*F*_(1,52)_ = 186.4, *p* < 1.0 × 10^−14^), with significant interaction (*F*_(1,52)_ = 29.42, *p* = 1.5 × 10^−6^; Fig. 4D). Taken together, these findings reveal a specific neuronal scramblase is required for, and elevated scramblase increases, critical period experience-dependent synapse pruning.

### Glial insulin receptors drive experience-dependent glial infiltration of synaptic glomeruli

Glial projections infiltrate the antennal lobe VM7 synaptic neuropil in response to olfactory experience during the critical period in a dose-dependent mechanism^13,16,37^. Given that phosphatidylserine is a membrane ligand working at the cell-contact interface, we next sought to identify a long-distance signaling mechanism capable of recruiting glial projections to the Or42a neuron synaptic glomeruli with critical period EB experience^1^. For glial clearance of *Drosophila* brain developmentally-transient neurons and debris following injury, insulin receptor (InR) signaling has been demonstrated to recruit glia for neuronal phagocytosis^25,28,29^. However, glial InR roles in the critical period, or in any form of experience-dependent synaptic pruning, have not been explored^1^. We hypothesized that glial InR signaling is required for targeted synaptic neuropil infiltration and synapse pruning in response to critical period EB experience. An *Or42a* receptor fusion with the mCD8::GFP membrane marker (*Or42a*-GFP) was used together with *repo*-Gal4 driven mCD8::RFP membrane marker in glia (*repo*-RFP), to test glial infiltration of the VM7 synaptic glomeruli with critical period exposure to the oil vehicle compared to EB odorant experience. There is a single conserved *Drosophila* insulin receptor (InR)^46^which has been shown to mediate signaling^25,28,29^. To test glial InR signaling requirements, *repo*-Gal4 was used to target UAS-*InR* RNAi to glia compared to the driver alone control in the oil vehicle and EB odorant experience conditions.

**Fig. 5:**
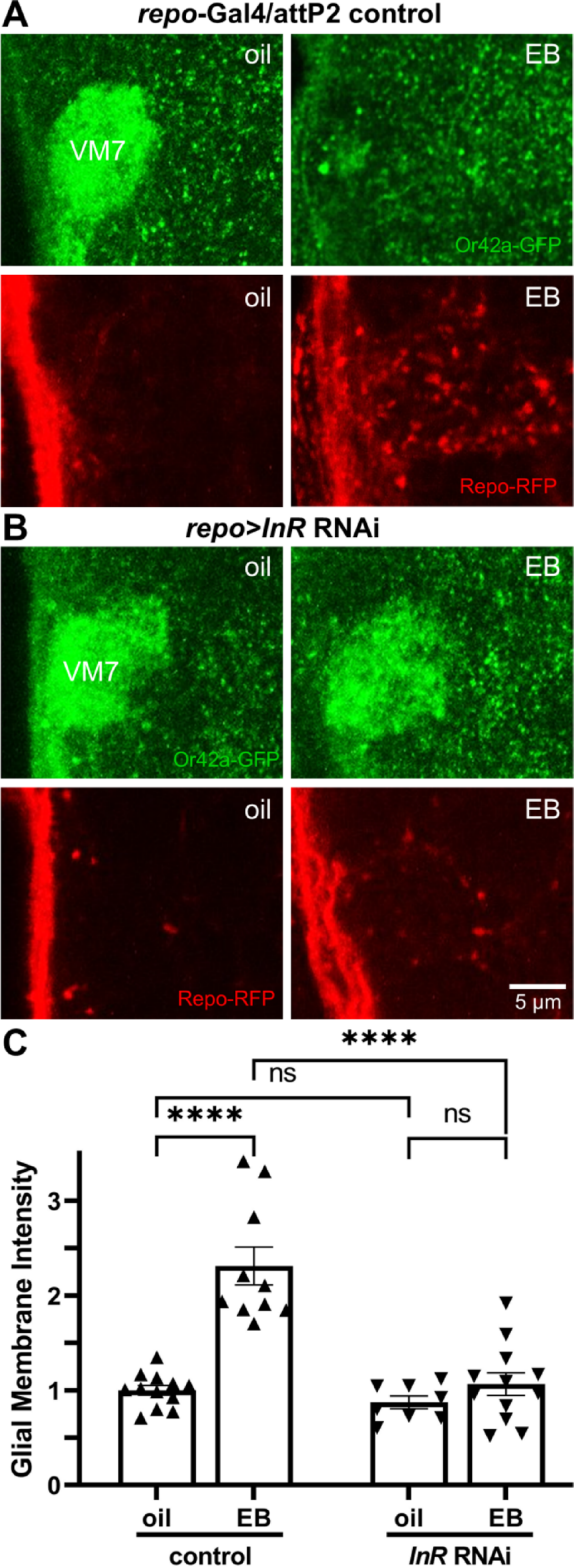
Glial insulin receptors drive experience-dependent glomerulus infiltration. (**A**) Representative images of Or42a neurons innervating the VM7 synaptic glomerulus (*Or42a*-GFP, green, top) and the glial *repo*-Gal4 driven UAS-mCD8::RFP membrane marker (*repo*-RFP, red, bottom) following 24-hour critical period exposure to the oil vehicle (left) or EB odorant (right). Normal experience-dependent innervation pruning occurs (top, right, green) with robust glial infiltration (bottom, right, red). (**B**) Representative images under the same conditions as above with *repo-*Gal4 driven *insulin receptor* (*InR*) RNAi targeted to glia (repo > *InR* RNAi, right). Experience-dependent innervation pruning fails (top right, green) with an absence of glial infiltration into the VM7 synaptic glomerulus (bottom right, red). Scale bar: 5 μm. (**C**) Normalized quantification of glial membrane fluorescence intensity levels in the *repo*-Gal4/+ driver control (left) compared to glial-targeted *InR* RNAi (right). Paired 24-hour critical period exposure to the oil vehicle (left) and EB odorant (right). Scatterplots show all the data points and the mean ± SEM. Significance is indicated at *p* ≤ 0.0001 (****) or as not significant (*p* > 0.05; ns).

Control animals exposed to oil vehicle show little detectable glial penetration into the antennal lobe VM7 synaptic glomeruli (Fig. 5A, left). In sharp contrast, glial projections strongly infiltrate following 24-hour critical period (0–1 dpe) EB exposure (Fig. 5A, right). Quantification of glial membrane fluorescence intensity in VM7 glomeruli normalized to the oil vehicle control (1.0 ± 0.052, *n* = 12) shows a significant elevation with EB experience (2.313 ± 0.201, *n* = 10; *q*_(38)_ = 10.81, *p* < 2.0 × 10^−8^; Fig. 5C, left). Thus, circuit-localized glial infiltration is targeted in response to critical period input. In comparison to *repo*-Gal4/+ driver controls with little detectable glia in the VM7 synaptic glomerulus (Fig. 5B, left), the glial-targeted *InR* RNAi is totally indistinguishable, lacking EB experience-induced glial projection infiltration (Fig. 5B, right). Quantification of the glial membrane fluorescence normalized to oil vehicle shows no significant difference in *InR* RNAi with oil vehicle (0.874 ± 0.067, *n* = 8) and no significant change with critical period EB experience (1.065 ± 0.119, *n* = 12; *q*_(38)_ = 1.481, *p* = 0.723; Fig. 5C, right). A two-way ANOVA (2 × 2) with Tukey’s multiple comparison post-hoc test comparing glial infiltration in both genotypes in vehicle relative to EB experience shows significant effects for genotype (*F*_(1,38)_ = 29.93, *p* = 3.0 × 10^−6^) and odorant experience (*F*_(1,38)_ = 35.91, *p* = 5.8 × 10^−7^), as well as the interaction between them (*F*_(1,38)_ = 19.93, *p* = 7.0 × 10^−5^; Fig. 5C). Taken together, these findings indicate that glial insulin receptor signaling drives experience-dependent targeted glomeruli glial infiltration during the early-life critical period.

### Glial insulin receptor function can bidirectionally determine critical period synapse pruning

**Fig. 6:**
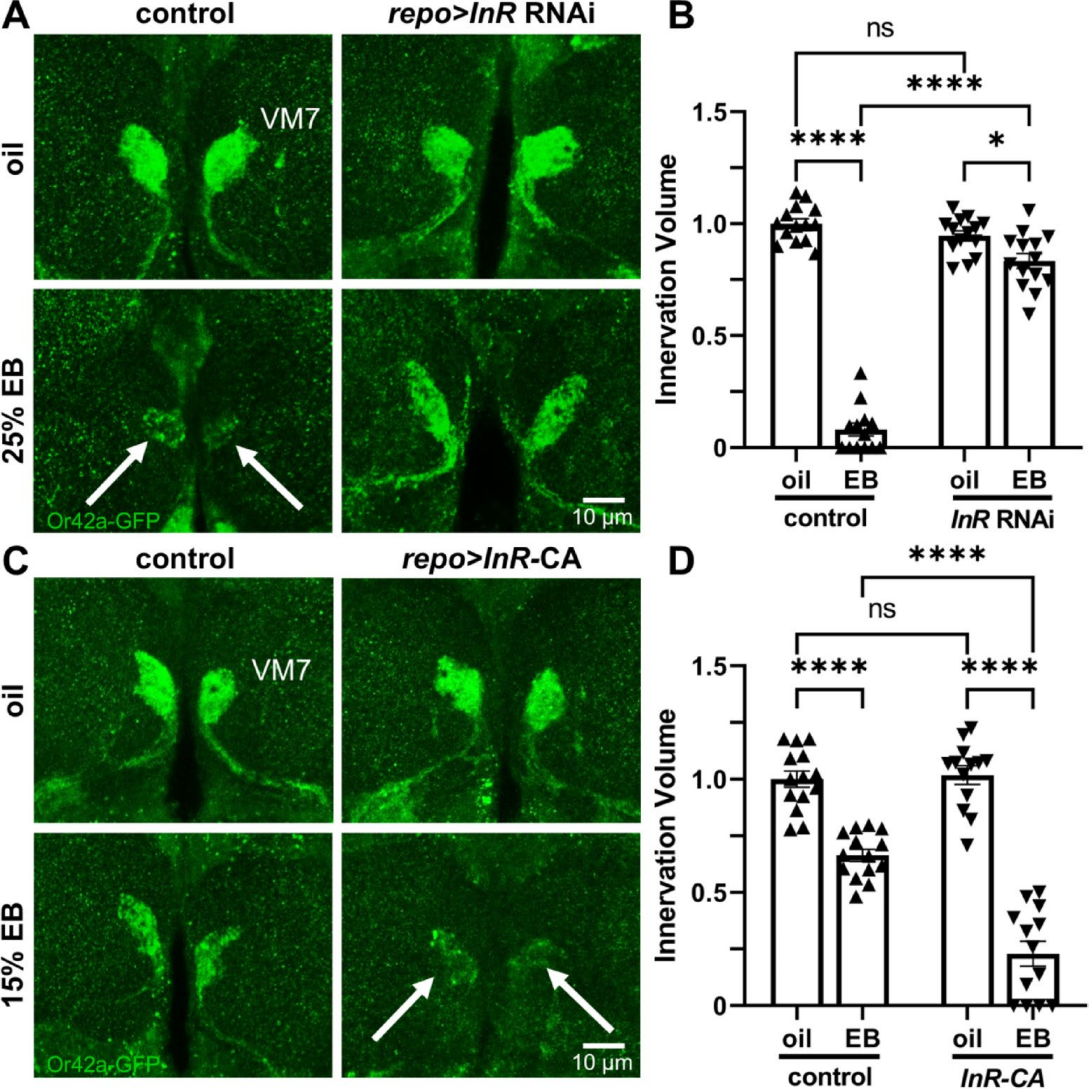
Glial insulin receptors bidirectionally control critical period pruning. (**A**) Representative images of Or42a neurons innervating the VM7 synaptic glomeruli (*Or42a*-GFP, green) following 24-hour critical period exposure (0–1 dpe) to the oil vehicle (top) or 25% EB odorant (bottom). The glial driver control (*repo*-Gal4/+) shows normal experience-dependent synaptic glomeruli pruning (left, bottom, arrows), which is strongly impaired by *repo*-Gal4 glial-targeted *InR* RNAi (*repo* > *InR* RNAi, right). Scale bar: 10 μm. (**B**) Quantification of the Or42a OSN innervation volume in VM7 glomeruli in all conditions, normalized to the oil vehicle control condition. (**C**) Representative images under the same conditions as above, except the oil vehicle (top) is compared to a reduced 15% EB exposure (bottom). The glial driver control (*repo*-Gal4/+, left) is compared to glial-targeted *repo*-Gal4 driven UAS-*InR* constitutively-active (CA) construct (*repo > InR-*CA, right). At 15% EB, the reduced Or42a OSN synaptic glomeruli pruning (left) is exacerbated by activating InR signaling in glia (right, bottom, arrows). Scale bar: 10 μm. (**D**) Quantification of the Or42a OSN innervation volume in VM7 glomeruli in all conditions, normalized to the oil vehicle control. Scatterplots show all the data points and mean ± SEM. Significance is indicated as *p* ≤ 0.05 (*), *p* ≤ 0.0001 (****), or not significant (*p* > 0.05; ns).

We next wanted to directly test the glial InR requirement in critical period pruning, using both glial-targeted *repo-*Gal4 *InR* RNAi and a constitutively active *InR* (*InR*-CA)^28^ compared to matched transgenic controls. In the latter test, the EB concentration was lowered to 15% to assess exacerbation of synapse pruning (as in Fig. 4). In the driver control (*repo*-Gal4/+), the oil vehicle condition shows the expected normal VM7 synaptic glomeruli innervation, and critical period (0–1 dpe) 25% EB experience causes strong synaptic pruning (Fig. 6A, left, arrows). Glial-targeted *InR* RNAi significantly inhibits this experience-dependent pruning (Fig. 6A, right column). Tukey’s multiple comparison tests normalized to the oil vehicle driver control innervation volume (1.0 ± 0.022, *n* = 14) reveals significant pruning with critical period 25% EB exposure (0.080 ± 0.029, *n* = 13; *q*_(51)_ = 33.86, *p* < 1.0 × 10^−14^; Fig. 6B, left). In contrast, glial-specific *InR* RNAi is similar to driver controls with the oil vehicle (0.946 ± 0.022, *n* = 14), and shows only slight Or42a innervation volume loss following 24-hour critical period 25% EB exposure (0.833 ± 0.033, *n* = 14; *q*_(51)_ = 4.203, *p* = 0.023; Fig. 6B, right). A two-way ANOVA (2 × 2) with Tukey’s multiple comparison post-hoc test comparing the innervation volumes shows significant effects for both genotype (*F*_(1,51)_ = 168.8, *p* < 1.0 × 10^−14^) and critical period odorant (*F*_(1,51)_ = 367.5, *p* < 1.0 × 10^−14^), with a significant interaction (*F*_(1,51)_ = 225.3, *p* < 1.0 × 10^−14^; Fig. 6B). These findings demonstrate that glia utilize insulin receptors for experience-dependent critical period pruning.

To test if increasing glial insulin receptor signaling could exacerbate pruning, we next tested glial-targeted *InR*-CA in the lowered 15% EB experience condition to allow the assay of potentially elevated synaptic glomeruli pruning. In transgenic driver controls (*repo*-Gal4/+), the oil vehicle shows normal Or42a OSN innervation, whereas the lower 15% critical period EB experience results in the expected dose-dependent decrease in synaptic glomeruli pruning (Fig. 6C, left). In contrast, constitutive-activation of glial InRs strongly increases EB experience-dependent innervation pruning (Fig. 6C, right, arrows). Quantification of innervation volume normalized to the oil vehicle control (1.0 ± 0.036, *n* = 14) reveals weaker 15% EB critical period experience drives significant smaller volume reduction (0.664 ± 0.027, *n* = 14; *q*_(50)_ = 8.447, *p* = 1.4 × 10^−6^; Fig. 6D, left). In comparison to *InR*-CA animals in oil vehicle (1.02 ± 0.041, *n* = 13), there is much greater Or42a OSN innervation pruning in the 15% EB experience condition (0.230 ± 0.055, *n* = 13; *q*_(50)_ = 19.11, *p* < 1.0 × 10^−14^; Fig. 6D, right). A two-way ANOVA (2 × 2) comparing innervation volumes reveals significant effects for both genotype (*F*_(1,50)_ = 26.38, *p* = 4.6 × 10^−6^) and odorant exposure (*F*_(1,50)_ = 192.5, *p* < 1.0 × 10^−14^), with a significant interaction term (*F*_(1,50)_ = 31.21, *p* = 9.5 × 10^−7^; Fig. 6D). Overall, these findings demonstrate that glia use insulin receptor signaling for experience-dependent synaptic glomeruli pruning, with a rate-limiting role dependent on the strength of sensory input during the critical period.

## Discussion

This study tests neuron-to-glia intercellular signaling mechanisms orchestrating the experience-dependent pruning of brain synaptic glomeruli by glia during an early-life olfactory critical period in juvenile *Drosophila*. This powerful genetic model enables the systematic genetic dissection of dose-dependent and temporally-restricted brain circuit synapse connectivity optimization^1^. We find critical period experience drives membrane phosphatidylserine (PS) externalization in target synaptic glomeruli in a dose-dependent mechanism (Fig. 1). We show this PS signaling plays a vital role in critical period experience-driven synapse pruning (Fig. 2). We use a nonallelic noncomplementation analysis^25,39,40^ to reveal a mechanistic link between neuronal PS synthesis and glial Draper/MEGF10 receptor-mediated synapse phagocytosis (Fig. 3). We discover that neuronal *subdued* PS scramblase^27^ is required for, and also that mammalian homologue overexpression enhances, critical period experience-driven synapse pruning (Fig. 4). We find glial insulin receptor signaling drives synaptic neuropil infiltration phagocytosis (Fig. 5), with activation limiting the experience-dependent synapse pruning (Fig. 6). Taken together, these results show neuron-to-glia intercellular signaling mechanisms that are both experience dose-driven and temporally-restricted to recruit glial projections for targeted synaptic glomeruli phagocytic pruning by glia in order to sculpt olfactory circuit connectivity dependent on the early-life critical period sensory environment^1,13,15^.

The new *Drosophila* brain connectivity maps^37,47,48^ reveal exact olfactory sensory neuron-to-projection neuron synapse linkages within antennal lobe glomeruli (Fig. 1A), but fail to consider experience-dependent remodeling. VM7 synaptic glomeruli innervated by EB-responsive Or42a neurons are pruned in response to EB odor experience in a tight temporally-restricted mechanism (Fig. 1B). This pruning mechanism is Or42a-specific and can be induced with optogenetic activation of Or42a OSNs in the absence of odor^49^. However, different OSN classes have varying responses to critical period experience^5,50^. For example, DM5 innervation expands in response to the same EB exposure^11^. It is not known why responses differ, particularly between neurons responding to the same odor, and it remains to be determined how the full glomerular map responds to timed critical period experience^8,11^. More complex odorant environments may well elicit synergistic or antagonistic synaptic remodeling outcomes in response to critical period experiences^10,51^. Behaviorally, OSN synaptic remodeling likely represents a mechanism to adapt juveniles to the odorant environment^10,50,51^. In this case, pruning of OSN-PN synapses should drive behavioral adaptations^37^. However, the effect of EB-induced critical period pruning on later behavioral odorant preferences, or changes in other OSN connectivity, remains to be tested. In VM7 glomeruli, we find EB experience drives dose-dependent PS lipid externalization in synaptic glomeruli membranes (Fig. 1C), indicating a circuit-localized signaling mechanism. Other OSN-specific odorants could also be tested to assess their differential neuron-to-glia signaling mechanisms in critical period synapse remodeling. More generally, future work needs to identify the range of experience dose-dependent and temporally-restricted intercellular signaling pathways that mediate all critical period synaptic remodeling, including synaptogenesis^52^.

We find phosphatidylserine (PS) production is required for experience-dependent synaptic glomeruli pruning in the critical period (Fig. 2). This pruning mechanism occurs only during the critical period and is completely reversible only in the critical period^11,12^. PS signaling is implicated in axonal clearance and synapse elimination^20,23,53–55^and PS externalization is necessary for both hippocampal and dorsal lateral geniculate nucleus synapse refinement during mammalian critical periods^22^. Our work indicates a conserved mechanism. We were unable to assess *phosphatidylserine synthase* (*pss*) null mutants due to their embryonic lethality^34^. Thus, we used Or42a OSN-targeted knockdown with *pss* RNAi while also removing one *pss* copy to test neuron-specific loss of PS production (Fig. 2). This genetic combination results in a striking impairment in experience-dependent synaptic glomeruli pruning. Consistent with previous reports^34^loss of *pss* in this combination does not result in any obvious behavioral alteration, although behavior was not subject to study. Moreover, the combined use of *pss* mutants to generate a viable, stronger loss-of-function condition results in an even greater inhibition of the odorant experience-dependent synapse pruning (Fig. 2). In future work, it would be interesting to explore other means of perturbing the experience-driven PS exposure on local synaptic membranes. One fascinating experiment would be to target expression of milk fat globule-EGF factor 8 (MFG-E8), which has been shown to mask externalized PS exposure to impair PS-dependent glial phagocytosis^24,56,57^. We would predict that the targeted Or42a OSN expression of secreted MFG-E8 should block local PS signaling directing glial phagocytosis to prevent critical period synaptic glomeruli pruning.

Genetic nonallelic noncomplementation analysis has long been used as an in vivo interaction test for many synaptic signaling mechanisms^25,39,40^. A *trans*-heterozygous combination between *pss* and *draper* null mutants reveals an interaction between PS production and glial Draper/MEGF10 receptor phagocytosis in experience-dependent synaptic glomeruli pruning (Fig. 3). Consistently, other studies have shown direct binding between PS ligand and Draper receptor^24^and suggested this ligand-receptor interaction targets glial phagocytosis during neuronal pruning as well as other remodeling mechanisms^20,33,57^. It would be interesting to explore the exact nature of this binding interaction, and whether synaptic membrane PS binds directly to glial Draper/MEGF10 receptors in critical period experience-dependent synapse pruning, or by agency of some bridging molecule^1^. In particular, the *Drosophila* chemokine-like Orion has been shown to bind to PS and mediate PS-to-Draper receptor interaction in other glial phagocytosis contexts^33^. Interestingly, Orion has also been implicated as a separable neuron-to-glia signal during mushroom body γ axon pruning in *Drosophila* pupal metamorphosis, with direct signaling requirements for glial infiltration and subsequent phagocytosis^58,59^. This fascinating reported dual nature for the Orion chemokine-like molecule, acting as a PS bridge to the Draper/MEGF10 receptor and/or acting as a separable signal, should be explored in critical period experience-dependent synapse pruning.

We discover the endogenous *subdued* neuronal PS scramblase plays a vital role in experience-dependent synaptic glomeruli pruning (Fig. 4). Scramblases function to maintain lipid symmetry between plasma membrane bilayers, whereas ATPases utilize energy to drive asymmetry^60^. The *Drosophila* P4-ATPase ATP8A flippase has been shown to maintain PS in the membrane inner leaflet^20,61^assisted by the CDC50 chaperone^62^. Simultaneous manipulations of ATP8A and CDC50 can alter PS levels to disrupt PS-dependent pruning mechanisms^20^. In future work, this manipulation could be attempted to sequester PS in the inner leaflet and assess effects on synapse pruning. Or42a-targeted overexpression of TMEM16F, the mammalian Subdued homolog^27^potentiates experience-dependent synaptic glomeruli pruning (Fig. 4). Manipulations of opposing TMEM16F and CDC50 functions (e.g., TMEM16F OE + CDC50 OE) might buffer PS externalization^20^offering alternative avenues for future analyses. TMEM16F function is calcium-dependent^44^so EB experience-driven Or42a neuron activation may allow TMEM16F to override flippase function. This model is consistent with results showing PS externalization is dose-dependent (Fig. 1C, D). Different levels of odorant experience provide differing strengths of Or42a neuron activation, thereby resulting in more PS externalization. Such an activity-dependent scramblase mechanism could thus mediate experience dose-dependent levels of critical period synapse pruning.

Specific critical period odorant experience targets glial phagocyte infiltration into the activated synaptic glomeruli^13,16,37^. Critical period EB exposure directs glial projections specifically into VM7 glomeruli (Fig. 5). We recently discovered that glial phagocytes rearrange their F-actin cytoskeleton for infiltration phagocytosis during the early-life critical period^13^. Experience-dependent signaling via the glia Draper/MEGF10 receptor causes Basket (mammalian JNK) phosphorylation and translocation into the remodeling glial nucleus, where it promotes transcriptional upregulation of AP-1 target genes, such as the F-actin crosslinking signaling scaffold Cheerio (mammalian FLNA)^13^. We discover here that knockdown of glial insulin receptor (InR) signaling prevents this experience-dependent glial infiltration into the VM7 synaptic glomeruli (Fig. 5). Importantly, glial InR signaling has been shown to upregulate Draper/MEGF10 receptor expression within a STAT92E-dependent mechanism following axonal injury^28^. In the future, the fascinating intersection between glial InR and Draper signaling could be tested in critical period experience-dependent synapse pruning. In particular, Cheerio/FLNA expression driven by experience could be tested with glial InR knockdown and activation. We predict that the lack of targeted glial infiltration with InR knockdown is due to reduced Cheerio/FLNA regulatory function on the actin cytoskeleton^13^providing a mechanistic link between glial InR signaling and the Draper-Basket-Cheerio pathway.

We discover that targeted loss of glial InR signaling severely impairs critical period experience-dependent synaptic glomeruli pruning (Fig. 6). Glial InR signaling mediates the glial clearance of developmentally-transient apoptotic neurons^25^ and neural debris following injury^28,63^ but, to our knowledge, we are the first to directly link glial InR signaling to critical period experience-dependent synapse pruning. Since the glial InR knockdown did not absolutely block synaptic glomeruli pruning, it would be interesting to test the downstream components of the insulin signaling pathway using glial-targeted RNAi for Akt or STAT92E^28^. We have not been able to directly demonstrate the critical period experience-dependent upregulation of activated glial insulin receptors. Current studies suggest this may prove impossible, but future studies could attempt phosphorylated InR antibody labeling^25^or other methods such as a glial-targeted STAT92E reporter^28^. It may also be that parallel pathways independently drive experience-dependent glial infiltration phagocytosis, so screens are needed to test this possibility. We find glial InR constitutive activation increases experience-dependent synaptic glomeruli pruning, with elevated synapse elimination in response to lower levels of critical period experience (Fig. 6). Similar activated glial InR signaling elevates Draper receptor expression in a *Drosophila* injury model^28^and it is possible that increased Draper receptor function is responsible for exacerbated synapse pruning from critical period experience. In any case, glial InR signaling clearly limits experience-dependent synapse elimination.

Glial phagocytosis is essential for the proper pruning of supernumerary synapses during brain circuit maturation^64,65^ and adult plasticity^66,67^. This precise, targeted synapse elimination process must be very tightly controlled through neuron-to-glia intercellular communication^1^. *Drosophila* injury models have provided indirect means to identify such intercellular signaling mechanisms, as injured neurons provide signals to recruit activated glial phagocytes for debris clearance^28,68^. Indeed, the signaling pathways explored here were discovered in just this way, proving their relevance to other glial phagocytosis mechanisms. Mammalian studies have validated conserved signaling functions, including PS and MEGF10, but have also revealed additional pathways, such as neuronal release of fractalkine (CX_3_CL1) and C1q presentation^22,64,69^. Future studies testing the growing list of putative neuron-to-glia signaling pathways are needed to provide additional insights into the multi-step cascade mechanisms driving glial infiltration phagocytosis^1^. In addition to *Drosophila* studies, mouse astrocytes have been shown to modulate critical period synapse refinement with diverse signaling mechanisms including adenosine, D-serine, NMDA receptor, and GABAergic signaling^70–74^. Such mechanisms could be examined in our *Drosophila* model, including astrocyte-like glial regulation of the extracellular matrix, potentially providing a means for controlling glial projection infiltration into the neuropil. Likewise, glia have already been shown to control critical period timing^6^ including the manipulated ability to re-open critical period-like plasticity^15^so employing the signaling mechanisms discovered here could provide an additional avenue to enable re-opening of critical period-like remodeling capabilities. Our *Drosophila* model provides a means to test conserved neuron-to-glia signaling mechanisms, and discover new pathways directing experience-dependent synapse pruning during the temporally-restricted critical period.

## Materials and methods

### Drosophila genetics

All stocks were reared at standard 25 °C on standard cornmeal/agar/molasses fly food. The genetic background control line for the Harvard Transgenic RNAi Project (TRiP) lines is the P{CaryP}attP2 third chromosome insert on the *y[1]v[1*] TRiP background. The genetic background control for mutants is *w*^*1118*^, to which all the genetic lines have been back-crossed^75^. The transgenic drivers are Or42a OSN-specific *Or42a-*Gal4^76^, pan-glial *repo*-Gal4^77^, and astrocyte-like glial *R86E01*-Gal4 (BDSC #45914). The MApHS reporter line is *w*^*1118*^; *TM6B*,* Tb/UAS-MApHS*, the *LactC1C2*GFP reporter line is *w*^*1118*^; *CyO*,* Wee-P/UAS-GFP-LactC1C2*, and the *TMEM16F* over-expression (OE) line is *w*^*1118*^; *CyO*,* Wee-P/UAS-TMEM16F*^20^. The Vienna *Drosophila* Resource Center (VDRC; Vienna, Austria) responder lines used are: UAS-*pss* RNAi (V#5391) and UAS-*subdued* RNAi (V#37472). The Bloomington *Drosophila* Stock Center (BDSC; Indiana University, Bloomington, IN, USA) responder lines used are: UAS-*InR* RNAi (BDSC #35251) and UAS-*InR-CA*(del) (BDSC #8248). The global mutant lines used are: *draper*^5^ deletion null mutant (BDSC #67033), *pss*^1^ deletion null mutant^34^*pss*^15^ (BDSC #22115) and *pss*^32^ hypomorphic mutants (BDSC #11632)^34^. All genetic crosses were done using standard *Drosophila* techniques, with a combination of visible markers and GFP balancers. All studies were done on adults staged in days post-eclosion (dpe) at 25 °C.

### Odorant exposure

Oil vehicle and ethyl butyrate (EB) exposures were done as previously reported^13–16^. Briefly, dark pupae (4 days after puparium formation at 25 °C) were staged based on genotype into separate vials capped with a fine stainless-steel. For critical period trials, the vials were placed into an airtight 3700 ml Glasslock container with either 1 ml mineral oil (vehicle control) or EB odorant dissolved in mineral oil (Sigma-Aldrich; 5–25% v/v EB) in 1.5 ml microcentrifuge tubes secured in the middle of odorant chambers. Both types of odorant chambers were placed in temperature-controlled, humidified incubators at 23 °C, maintained on 12 h light/dark cycles. After 4 h in the incubators, newly-eclosed flies were rapidly transferred to clean tubes with fresh vials and kept in the odor chambers for an additional 20 h (24 h total; 0–1 dpe) before being immediately processed for immunocytochemistry. For mature adult trials, newly-eclosed flies were maintained at 25 °C for 1 week, and then exposed to either oil vehicle or EB odorant as above for 24 h (7–8 dpe) before being immediately processed for immunocytochemistry.

### Brain labeling

Staged *Drosophila* brains were dissected in phosphate-buffered saline (PBS) at room temperature (RT), and then fixed in 4% paraformaldehyde (PFA) + 4% sucrose in PBS (pH 7.4) with constant circular rotation for 30 min at RT^14^. Fixed brains were next quickly washed 3X in PBS. After washing, the brains were placed into a blocking solution (1% bovine serum albumin (BSA) + 0.5% normal goat serum (NGS) in PBS + 0.2% Triton-X 100; PBST) for 1 h with constant rotation. Brains were incubated at 4 °C overnight with primary antibodies in blocking solution (0.2% BSA, 0.1% NGS in PBST), and then washed 3X in PBST for 20 min with constant rotation. Primary antibodies used: chicken anti-GFP (Abcam, ab13970; 1:1,000) and rat anti-RFP (ChromoTek, 5f8; 1:1,000). After washing 3X in PBST for 20 min, the brains were incubated with secondary antibodies in blocking solution for 2 h at RT, followed by 3X final washes with PBST for 20 min. Secondary antibodies used: 488 goat anti-chicken (Invitrogen, A11039; 1:250) and 546 goat anti-rat (Invitrogen, A11081; 1:250). Brains labeled for externalized phosphatidylserine (PS) were processed exactly as above, except all detergent was excluded.

### Confocal analyses

Labeled brains were mounted in Fluoromount-G Mounting Medium (00-4958-02) under a glass coverslip (No. 1.5 H, Carl Zeiss). Double-sided tape was used as a spacer between the brain and coverslip, with the slides sealed using clear nail polish (Expressie, Essie)^14^. All slides were imaged using a laser-scanning confocal microscope (Carl Zeiss LSM 510 META) at 1024 × 1024 resolution, and then projected using ZEN microscopy software. Low magnification images (Figs. 1B, 2, 3, 4 and 6) were taken using a 40x oil objective, and high-magnification images (Figs. 1D and 5) were taken with a 63x oil objective. Imaging settings were identical within all biological replicates in all experiments, and all images were blinded prior to analyses. For innervation volume measurements, ROIs were created around the maximal borders of VM7 glomeruli and the FIJI plugin 3D Objects Counter was used to quantify innervation volume^14^. For analyses of LactC1C2-GFP labeling and glial infiltration, an oval 150 × 150 pixel ROI centered on the VM7 glomerulus was created. The mean ROI fluorescence intensity was measured for the entire glomerulus volume Z-stack (~ 20 slices) and averaged to quantify intensity.

### Statistical tests

All statistical analyses were performed using GraphPad Prism software (v9.0). All data sets were analyzed using a D’Agostino-Pearson normality test. All normal data sets were analyzed using parametric tests. For data comparing 1 genotype and ≥ 2 odorant conditions, a one-way ANOVA was used, followed by Tukey’s multiple comparison tests to analyze both genotypes and treatment conditions. For data comparing ≥ 2 genotypes and ≥ 2 odorant conditions, a two-way ANOVA was used with odorant exposure and genotype as independent variables, followed by Tukey’s multiple comparison tests to analyze both genotypes and treatment conditions. All figures show scatterplots with all data points, as well as the mean ± SEM. Significance is shown as *p* < 0.05 (*), *p* < 0.01 (**), *p* < 0.001 (***), and *p* < 0.0001 (****), with *p* > 0.05 indicated as not significant (ns).

## Data Availability

All original data analyzed during this study are publicly available at the Harvard Dataverse.
